# “Insane Hospital Supervision,” and the State Board of Public Charities of Illinois

**Published:** 1886-07

**Authors:** Fred. H. Wines

**Affiliations:** Springfield


*To the Editors of the* CHICAGO MEDICAL JOURNAL AND EXAMINER:

*Gentlemen.*—A pamphlet copy of an article on Insane Hospital Supervision, reprinted from the CHICAGO MEDICAL JOURNAL AND EXAMINER for April, has come under my notice —not having been sent me by the author or publisher, nor indeed sent to me by any person—which is in substance an assault upon the State Board of Public Charities of Illinois, for failing to do what the writer conceives to have been its duty, in the matter of the recent investigation by it of the management of the Cook County Insane Hospital.

No member of that board will, I am sure, object to any fair or legitimate criticism of its official action.

I am constrained, however, by my own sense of fairness, not being myself a member of the board, to ask you to print in your May number the following explicit and categorical denial of some of the misstatements contained in the article referred to. Dr. Spitzka may perhaps be excused for his errors, on the ground that he does not reside in Illinois, was not present at the investigation, can not have seen the testimony (which has not been printed), and is therefore in the favorable condition of mind for a reviewer described by Sydney Smith, when he said that he never read a book before reviewing it, lest it should prejudice his judgment.

(1). The State Board did not, in its last biennial report, "commend the Cook County Insane Hospital management as equal to the best State asylum," nor speak of it in "eulogistic terms," nor "laud the superintendent for reducing restraint." It said:

"In relation to restraint, Dr. Spray remarks," etc. It merely quotes his words, without comment of any sort. It does not even assent to their truth.

It also said:

"The institution is conducted in the same manner as are the State hospitals, and the same regard is had to cleanliness, ventilation and the care and comfort of patients. In most respects, this asylum compares favorably with our State institutions. In only one ward was the hospital odor found to be offensive, and steps were being taken to remedy the matter."

The asylum was visited in August, and the report does not purport to give anything but the appearance of the institution to the eye of the observer. It was precisely what it is said to have been. It was clean, it was well aired, and the patients seemed to be comfortable. In these respects, it compared favorably with our State institutions. It was conducted "in the same manner as" the State hospitals; that is to say, not in the same manner as an ordinary county almshouse. It had the organization of a hospital, with a medical superintendent, residing in the institution, assistant physicians, matron, a clerk, a druggist, supervisors, attendants on the wards, night watches, etc., which almshouses as a rule have not. But this is far from saying that the management was equal to the best State asylum." The board had repeatedly expressed its opinion of the management in uncomplimentary terms, particularly in the report of 1878. It has uniformly and persistently held up the system of appointments and control to public opprobrium, when not a newspaper in Chicago would reprint its comments.

(2). The Chicago Medical Society and the Citizens' Association did not, in 1884, "request the board to unite with them in investigating" the institution, nor did either of them do so.

(3). The governor did not, in 1886, "order the board to make an investigation." On the contrary, he declined to issue any order, for the reason that he had no power, under the statute, to do anything of the kind.

(4). The board was not "desirous to screen the administration during 1883–4," and it did not screen it. If the board screened it, then Dr. Spitzka has also screened it. Nearly every statement made by him as to the condition of the asylum is quoted (for the most part *ipsissimis verbis*) from the report made by the board, and without giving credit (except in isolated instances) for his quotations. Either by accident or design, he has so used language as to convey the false impression that he is revealing what the board endeavored to conceal.

(5). The board did not "enforce the legal rules of evidence against Dr. Kiernan" and "fail to enforce them in his favor." Judge Grimshaw, who presided, a lawyer of half a century's experience and of the highest standing, repeatedly stated that in an investigation of this character (which was not a trial) it would be impossible to enforce strict legal rules of evidence; and that he was disposed to allow to both sides whatever degree of latitude might be requisite, in order to ascertain to the satisfaction of the board all the facts in the case, but that he would be obliged to keep the witnesses as close to the point at issue as possible. It was his manifest desire to treat both sides with absolute impartiality, and I venture to say that not a disinterested spectator who was present at any session of the investigation will deny this assertion.

(6). The board did not "refuse to allow Dr. Kiernan to introduce any direct evidence after the rebuttal evidence of the accused." It considered that he had substantially proved his charges; the defence, if it can be so called, had virtually broken down: the public was weary of the whole matter; and no further evidence was offered by him, nor was any required.

(7). The board did not "refuse to render him assistance" in procuring witnesses. It gave him all the assistance in its power. The law does not confer upon it power to summon witnesses, and of course it cannot compel their attendance. It did issue subpœnas, on its own responsibility, for every witness named by him, but having no officer at its command to serve the same, it gave them to Dr. Kiernan to serve, which was all that it could do.

(8). The board did not "refuse at first," or at any other time, "to allow him legal aid." It may have expressed the opinion that he needed none. What the force of the expression "at first" may be, I have no idea. He had precisely the same assistance from the attorney for the Citizens' Association at first, that he had at any time during the investigation.

I am impelled to ask how Dr. Spitzka assumes to know the details of a trial or investigation at which he was not present, and on what authority he bases charges so utterly flimsy and unsupported.

(9). This is the first time that I at least have heard that the request for an investigation came from a "Chicago bar committee"—who were they?—or from "the editors of influential papers and leading citizens," other than the Citizens' Association, the Woman's Club and the Chicago Medical Society.

Into any examination of the other inaccuracies of statement into which Dr. Spitzka has fallen, when he has undertaken to go outside of the record, I have neither the time nor the inclination to enter.

His conclusions are vitiated, first, by the want of connexion between the conclusion and the premises; second, by the erroneous character of the premises which he assumes; and third, by his evident desire to turn this investigation to special account as an argument in favor of a preconceived conclusion in favor of State commissions in lunacy.

I may be permitted to remark that the State Board of Charities of Illinois, which he assails, has declared itself in favor of a State commissioner in lunacy, and that a bill embodying this proposition has been introduced in the legislature of Illinois, with the sanction of its approval and endorsement. But the legislature would not hear to it. He is fighting his allies, instead of his enemies.

Both Dr. Kiernan and Dr. Spray seem to think—naturally enough, perhaps—that THEY were the parties on trial before the bar of public opinion. The State commissioners utterly failed to disabuse their minds of this impression, and had an arduous task to keep the investigation, which lasted for more than a week, from degenerating into an interminable wrangle over personalities. The real issue was not their individual responsibility for the existing condition of affairs (which it was plainly beyond the power of either of them to remedy), but the responsibility of the COUNTY BOARD. It was the county board which was on trial; and the verdict was one of condemnation. If this verdict is not in accordance with the evidence, what should have been the form of verdict rendered ?

The board had no wish to reward or punish, to help, or to injure, Dr. Kiernan, or Dr. Spray, or any one else: What it sought was the protection of *the insane.* It desired to find and apply a remedy. There is no remedy except in legislation. The legislature does not know Dr. Kiernan or Dr. Spray, nor care for either of them, except as citizens. It knows the county board; it has power over the county board; it can deal with it. The State commissioners have, in my judgment, laid the foundation for a successful appeal to the legislature at its next session for some remedial legislation.

Such attacks as those of Dr. Spitzka are calculated to injure the cause of the insane, rather than to benefit it. He simply does not understand what he is talking about.

Very respectfully,

FRED. H. WINES.

Springfield.

